# Genetic Diversity among Methicillin-Resistant *Staphylococcus aureus* in Malaysia (2002–2020)

**DOI:** 10.3390/tropicalmed7110360

**Published:** 2022-11-08

**Authors:** Hassanain Al-Talib, Syahirah Samsudin, Ariza Adnan, Chandrika Murugaiah

**Affiliations:** 1 Department of Medical Microbiology and Parasitology, Faculty of Medicine, Universiti Teknologi MARA (UiTM), Sungai Buloh 47000, Malaysia; 2 Institute for Medical and Molecular Biotechnology, Universiti Teknologi MARA (UiTM), Sungai Buloh 47000, Malaysia; 3 Department of Biomedical Sciences and Therapeutics, Faculty of Medicine and Health Sciences, Universiti Malaysia Sabah, Jalan UMS, Kota Kinabalu 88400, Malaysia

**Keywords:** MRSA, genotyping, MLST, PFGE, SCC

## Abstract

Background: Methicillin-resistant *Staphylococcus aureus* (MRSA) is a common organism seen in both healthcare-associated and community-associated infections worldwide and in Malaysia over the past two decades. The aim of this review is to provide a firsthand documentation of all MRSA strains prevalent in the Malaysian population from 2002 to present and briefly describe the changing patterns. Methods: Electronic and manual intensive literature searches were conducted between 2002 and 2020, addressing issues directly related to patients and published in the English language were selected. Results: The literature search retrieved a total of 2217 articles and abstracts of 27 articles. The search yielded a total of 24 articles on genotyping of MRSA in Malaysia. The study found that MRSA strains were mostly genetically related and resulted in the predominant MRSA clones that caused active infections. Thirty-six different sequence types (ST) were recorded. The highest rates of STs detected were ST239 (52.6%), ST1 (47.4%), and ST22 (42.1%). The majority of studies showed that both SCCmec types III and IV were the most common SCCm type in Malaysia, followed by SCCmec type V (57.9%). Conclusions: Both Brazilian (ST 239 IIIA) and Hungarian (ST 239-III) MRSA strains were detected in Malaysia. PFGE remains the best method for comparing MRSA strains. However, whole-genome sequencing has a promising chance to replace PFGE in the future.

## 1. Introduction

Methicillin-resistant *Staphylococcus aureus* (MRSA) is a worldwide pathogen leading to healthcare-associated infections and is commonly resulting in significant mortality, morbidity, and an increase in hospital stay cost burden [1]. The genetic components of *S. aureus* are mostly encoded on mobile genetic elements (MGE). They are usually defined as DNA fragments encoding a set of virulence and resistance factors that initiate their transition and incorporation into new host DNA [2]. The MGE in *S. aureus* include bacteriophages (phages), pathogenicity islands, plasmids, transposons, integrative conjugative elements (ICEs), integrons, and staphylococcal chromosome cassettes (SCCs) [3].

It is important to note that phenotypic methods such as antibiotic susceptibility testing did not differentiate between healthcare-associated MRSA (HA-MRSA) and community-associated MRSA (CA-MRSA) [4]. Although MRSA rates in general appear to be decreasing, infection rates within the local community are steadily increasing, which is a cause for concern [5]. However, transmission of antibiotic resistance in hospitals has blurred the distinction between CA-MRSA and HA-MRSA [6]. Hospital environments could act as potential reservoirs for transmission of MRSA encoding various antibiotic resistance and virulence genes. This was confirmed by molecular typing, showing that MRSA isolates from different surfaces within the same hospital (intraclonal spread) and between different hospitals (interconal spread) are genetically similar [7]. Rates of community- and hospital-acquired infections caused by ST239-MRSA have increased over the past two decades [8]. ST239 is a single-locus variant of ST8 and it is a globally disseminated HA-MRSA lineage characterized by resistance to erythromycin and ciprofloxacin [9]. In Southeast Asian countries, including Malaysia and Singapore, MRSA-ST239 containing SCCmec type III was the predominant clone in the mid-1980s, which was then replaced by MRSA-ST22-SCCmec type IV isolates in the early 2000s [10]. In Malaysia, the genetic diversity of circulating ST239 clones increased from 2007, and a subclone of ST239 emerged that contains a mobile arginine catabolism element (ACME) encoding genes associated with increased skin colonization [10]. MRSA is often associated with clonal spread and diversity, and molecular typing methods have therefore been used for epidemiological surveillance [11]. In Malaysia, the clonal relationship between MRSA isolates was analyzed using various methods, including multilocus sequence typing (MLST) [12], staphylococcal cassette chromosome mec (SCCmec) [13], pulsed-field gel electrophoresis (PFGE) [9] and staphylococcal protein A (spa) [14]. Data on molecular genotyping of MRSA isolates in Malaysia appear to be sparse compared with those published in the United States and Europe [15].

In Malaysia, the clonal diversity and exchange of MRSA strains in different hospitals changed within a short period of no more than eight years [16].

Awareness of the molecular epidemiology of MRSA will help reduce clonal spread between different hospitals, cities, countries, and even continents [17]. Knowledge of the genetic profile, dynamic epidemiological clonal variation, antibiotic resistance, and spread of similar MRSA strains is also critical to evaluate the efficacy of existing control measures, gain insight into the evolutionary mechanism, trace the source of infection, and limit spread.

An example of the global initiative to combat the spread of MRSA is the establishment of numerous MRSA surveillance networks such as the Asian Network for Surveillance of Resistant Pathogens (ANSORP) [18]. Certainly, it included antibiotic resistance and molecular background data from Malaysian hospitals. Malaysian MRSA antibiotic resistance prevalence is reported at the national level as part of the Malaysian National Surveillance of Antimicrobial Resistance (NSAR) [19].

Therefore, the aim of this paper is to provide a compilation of previously published data on MRSA infections in Malaysia. This will provide a more comprehensive understanding of the molecular epidemiological evolution and genotypic relatedness between different isolates. The four different typing methods used to study the relatedness of different MRSA strains are PCR-based methods, PFGE, restriction fragment length polymorphism (RFLP), and SCCmec typing; in addition, other sequence-based methods such as MLST and spa gene typing. The reproducibility of PFGE is considered higher compared to other techniques due to the standardization of laboratory techniques [20]. PFGE has also been used as a tool to monitor and track the transmission and spread of MRSA in hospitals in Malaysia [21]. Multilocus Sequence Typing (MLST) was introduced for microbial specification to allow faster and universal differentiation between species. MLST detection for MRSA is based on the sequencing of seven genes in the bacterial chromosome that are thought to be conservative [22]. Staphylococcal protein A (spa) typing is a sequence-based method that targets the X region of protein A encoded by the spa gene and contains polymorphic direct repeats [23]; Staphylococcal cassette chromosome mec typing (SCCmec) is based on the eleven types (I–XI) of SCC-mec depending on the arrangement of the mec complex, the ccr complex, and the incorporated plasmids type [24].

## 2. Materials and Methods

### 2.1. Bibliographic Databases

A literature search was conducted both electronically and manually. Electronic data and articles published in English were searched online in PubMed. In addition, a manual search was performed in the references of selected articles. A total of 2217 articles were retrieved from the literature search using the keywords “MRSA genotyping” and additional filters. Abstracts of previously published articles with titles related to this study were reexamined. Manuscripts demonstrating genetic diversity and/or genotyping or clonal similarity of MRSA in Malaysia were used as the selection criteria. A summary of the results of the literature search in a bibliographic database and common search engines is discussed in the following section.

### 2.2. Literature Search Method

Studies included in the review were selected according to the following criteria: Patients (P): patients from hospitals and community populations who were MRSA-positive; Intervention (I): use of different genotyping methods to categorize MRSA; Comparison (C): quantitative assessment of the distribution and variations of MRSA; Outcomes (O): Determination of the predominant MRSA strain (s) subtyping in Malaysia over 18 years; Study (S): Data analysis was then performed based on these search results. A specific search was performed in PubMed and Google Scholar using the terms “methicillin resistant *Staphylococcus aureus* (MRSA)”, “genotyping”, “clonal di-versity”, “DNA fingeprinting”, “pulsed-field gel electrophoresis (PFGE)”, “multilocus sequence typing (MLST)”, “staphylococcal cassette chromosome (SCCmec)”, “staphylococcal protein A (spa)”, and “Malaysia”. Only articles published between 2002 and 2020 that addressed issues directly related to patients and were published in English were selected. They were also categorized according to the MRSA genotyping method used. Research articles published in non-peer-reviewed journals and those that failed to cite the sources of the data, which may introduce bias, were excluded from this study.

## 3. Results

Abstracts of 27 articles on the study topics were reviewed. Searches of PubMed and Google scholar yielded a total of twenty-four articles on MRSA genotyping in Malaysia (Table 1). Of the twenty-four articles on MRSA genotyping, one study used PCR-RFLP typing of the coagulase gene (coa) and found that MRSA strains were largely genetically related and formed predominant MRSA clones that resulted in active infections [25]. Nineteen studies had used MLST. Thirty-six different sequence types (ST) were found in these studies. The highest rate of STs detected in previous studies were ST239 (52.6%), ST1 (47.4%), and ST22 (42.1%). In the last 18 years, nearly eighteen new STs were reported for the first time in Malaysia, including ST20, ST80, ST96, ST101, ST129, ST152, ST241, ST573, ST769, ST951, ST1137, ST1241, ST1283, ST1284, ST1285, ST1286, ST1287, and ST1288. The results are shown in Table 2.

For spa typing, it was targeted in 14 previous studies which showed 55 various spa types. Replicates between spa types varied from 1 to 6. The majority (31.6%) of spa types belonged to t037, t421, followed by t032, t0127 (26.3%). Nine SCCmec types were detected, namely, SCCmec type I, II, III, IIIA, IV, IVA, IVB, IVC and V. The majority (68.4%) of studies showed that both SCCmec type III and IV were the most common SCCmec type in Malaysia, followed by SCCmec type V (57.9%). The pvl gene test was performed in 18 studies to distinguish MRSA isolates (Table 1). The origin of MRSA isolates published in previous studies was HA-MRSA 23 (82%), CA-MRSA 9 (32%) and mixed MRSA from the hospital and community 4 (14.2%).

## 4. Discussion

The relatively high MRSA prevalence rate of 14.9% according to the Malaysian National Antibiotic Resistance Surveillance Report (2020) [19] indicates the need for rapid and sophisticated subtyping methods. In Malaysia, investigators used different techniques for MRSA genotyping, making it difficult to draw a clear conclusion about the predominant MRSA clones. So far, five major pandemic MRSA clones have been identified. These include the Iberian or ST 247-IA clone, the Brazilian or ST 239 IIIA clone, the Hungarian or ST 239-III clone, the New York/Japan or ST 5-II clone, and the paediatric or ST 5-IV clone [15,27,44]. Global MRSA strains such as ST239-SCCmec III, ST30 SCCmec IV, ST22-SCCmec IV, ST1-SCCmec IV, and ST5-SCCmec II were known to be circulating and spreading clones within and outside countries [33].

Unfortunately, both Brazilian and Hungarian MRSA clones (MLST ST239) have genetic characteristics that enhance their ability to form biofilms that promote adherence and subsequent invasion of human respiratory cells [45]. As in most Asian countries, the recorded studies in Malaysia between 2002 and 2020 showed that most MRSA strains belonged to the pandemic clones ST239-SCCmec III and IIIA and spa type t037. These clones were widely distributed in various teaching hospitals in Kuala Lumpur and several other states, and these isolates were resistant to the β-lactam antimicrobial agents; they also showed resistance to other antibiotics such as erythromycin (100%), cotrimoxazole (98.3%), clindamycin (88.3%), rifampicin, and mupirocin (6.7%) [12,15,29,40]. In Korea and Japan, most MRSA clones belonged to the ST5-SCCmec type II [27]. The high prevalence of the ST239-IIIA clone may be due to its greater spread in hospitals and communities. A previous study in Malaysia by Lim et al. (2012) showed a fluctuation in the predominance of ST239 subtypes over time. The predominant ST239-t037 clone decreased from 95% in 2003 to 66% in 2008, although there was a slight increase in the prevalence of ST239-t421 from 2% in 2003 to 18.6% in 2008 [34]. A combined analysis using MLST-spa-dru typing showed that the percentage of MLST239-t037-dt13d decreased from 49% in 2003 to 14% in 2008 and was replaced by MLST239-t037-dt13g [34]. A study conducted by Lim et al. (2012) found that both MRSA and MSSA strains have a combined MLST-spa type ST239-t037, suggesting that MRSA strains may have evolved from MSSA strains [45]. In addition, previous reports confirmed the association between MLST and certain virulence genes such as sea and ST -239; sec and ST -22; she and ST -1; seg and sei with ST -188, ST -1 [13,15]. A study by Song and colleagues (2011) in China found that both HA-MRSA (SCCmec I, II, and III) isolates are spreading in the community and CA-MRSA (SCCmec IV) isolates are spreading in healthcare settings in Vietnam, the Philippines, Taiwan, Hong Kong, Thailand, and Korea [46]. In Malaysia, Sit et al. (2018) found that SCCmec types IV and V (which predominates among CA-MRSA) were the cause of bacteremia at the University Malaya Medical Centre. The SCCmec type III was also found in the community [9]. This was in contrast to an earlier study by Mat Azis et al. (2017), in which they found that, among students in the Selangor district, nine out of ten CA-MRSA isolates belonged to SCCmec type I, which is isolated from HA-MRSA [39]. In addition, previous studies by Ahmad et al. (2006 and 2008) have shown that CA-MRSA strains in nine Malaysian hospitals have the MLST type ST30-SCCmec type IV [26]. However, the presence of MLST ST22 with SCCmec type IV, which is often a community-acquired clone, has signaling as they have converted to nosocomial pathogens. It is also known that ST22 generally circulates in Europe and belongs to the EMRSA-15 [47]. Ghaznavi-Rad et al. (2010) identified for the first time in a Malaysian hospital cases of infection with ST22 with SCCmec type IVh, with three different spa types (t4184, t3213, and t932). ST22, SCCmec IVh was previously reported in Portugal, Sweden, and Spain [15]. The spread of ST22 is of particular interest as this strain currently dominates infections caused by ST239 in Malaysia. Niek et al. (2019) found that the current majority of MRSA isolates in Malaysian hospitals belonged to ST22-MRSA-IV. This could be due to cross-border migration of people that would allow transmission of this infection on a global scale. Since SCCmec type IV is the smallest structural type of SCCmec, it is believed to be more transmissible and also more variable than other SCCmec types [40].

ST241-t363 is a new strain in Malaysia discovered in 2008 and associated with rifampicin and fusidic acid resistance [48]. Sit et al. (2018) have reported for the first time the presence of HA-MRSA clones ST152-SCCmec I, ST45-SCCmec V, and ST951-SCCmec V [9]. ST152 clones have been associated with sporadic disease in Central Europe, the Balkans, Switzerland, and Denmark. This reflected a high probability of worldwide transmission to Malaysia. In addition, all ST152 carried the pvl gene [49]. A study by Zarizal et al. (2018) revealed a diverse genetic background of nasal MRSA isolates, including 11 different spa types and a dominance of the t548 clone, which is associated with sequence typings ST5 and ST97 [37]. A previous study by Sit et al. (2017) reported the first appearance of MRSA clones such as ST508, ST5-SCCmec V, ST1-SCCmec IV, and ST1137-SCCmec IV in Malaysia [33]. In addition to ST 508, in 2017, Mat Azis and co-workers reported another three MRSA isolates belonging to ST88 and ST96 SCCmec type I [39]. A new ST573 clone reported by Lim et al. is genetically related to a previously reported ST1 clone with a single-point mutation in the pta locus. Similarly, this clone is also related to another MRSA clone (ST188) which was reported by Ghaznavi-Rad et al., in 2011. Both clones are from the same clonal complex, CC1, and they differed by 3-point mutations [34].

It is noteworthy that the clonal diversity of MRSA strains increases over time. For example, SCCmec in type IV has different sequences of ST1, ST5, ST6, ST22, and ST 1137, indicating that it is able to transfer its genetic sequence between genetic clones of *S. aureus* [33,50]. Comparable results have been reported in other studies on the presence of ST30, ST1179-SCCmec type IV, and ST1-SCCmec type V, possibly indicating the establishment of these clones with epidemic potential in Malaysian hospitals [15]. Conversely, a previous study has shown that MRSA strains are genetically related, whereas methicillin-sensitive *S. aureus* strains exhibit greater heterogeneity [45]. In addition, a study conducted by Thong et al. (2013) found that multidrug-resistant MRSA clinical isolates from the University of Malaya Medical Centre were genetically related, suggesting that only a few major clones of the species were involved in infection [29].

According to Lim et al. (2012), PFGE was found to be more discriminatory than MLST, PCR-RFLP of the coa gene and spa in subtyping the MRSA strains [45]. In addition, Simpson’s Index of Diversity (SID) showed greater diversity of MRSA strains by PFGE compared to MLST [9]. Nevertheless, PFGE can accurately describe ancestral strains of MRSA by combining PFGE with phenotypic and/or molecular characteristics of the strains, such as antimicrobial susceptibility, SCCmec types, PVL, and MLST, in an integrated algorithm platform [9]. On the other hand, the discriminatory ability of dru typing, especially in closely related MRSA ST239 strains such as Hungarian and Brazilian strains, confirmed its utility as a tool for epidemiological investigation of MRSA [28]. Furthermore, dru typing can also be used for additional differentiation between MRSA clones within the same SCCmec type, e.g., between SCCmec IIIa and III isolates. SCCmec IIIa isolates had nine dru types (dt1a, dt9w, dt15e, dt13j, dt13d, dt13h, dt13g, dt11aa, and dt13e), whereas seven types (dt13i, dt13d, dt14b, dt14d, dt12g, dt14c, and dt12h) were observed for SCCmec III [28].

Several notable studies have concluded that using both spa- and mec-associated dru typing methods can provide higher discrimination of ST239 clones. These can be further categorized into 7 spa types and 26 different dru types [28,34], which indicated that there are at least 7 subtypes circulating in a single hospital. Therefore, it appears that a combination of spa- and mec-associated dru typing is in par excellence with MLST and might even supersede MLST in subtyping MRSA. This is mainly because both spa- and mec-associated dru typing are faster, less labour intensive, and feasible. The discrepancy observed between MLST and spa could be due to the selection of target genes among the techniques and also could be due to the difference in discriminatory power of each technique [36]. However, the only disadvantage of the mec-associated dru typing method is that it is not able to distinguish the different ancestry of MRSA strains, nor can it be used for methicillin-sensitive *S. aureus* strains, as these lack the mecA gene [34]. Therefore, it is recommended that mec-associated dru typing be used in conjunction with another sequence typing method, such as spa typing, to classify MRSA strains.

## 5. Conclusions

Pandemic MRSA clones originating from Brazil (ST 239-IIIA) and Hungary (ST 239-III) are some of the strains already detected in communities in Malaysia. Also, transmission from HA-MRSA can often spread easily in the community, resulting in undocumented clonal spread and diversity. PFGE provides the best discriminability and reproducibility of results compared to other techniques. With the many techniques currently available, there is a definite need for a standardized method for identifying MRSA clones, such as whole-genome sequencing (WGS), which reveals the entire genome of target isolates and allows identification and characterization of MRSA.

## Figures and Tables

**Table 1:** Clonal characteristics of methicillin-resistant *Staphylococcus aureus* isolates in Malaysia from 2002 to 2020.

Setting	Year	Source of MRSA	Molecular Typing Methods	MLST Types	*Spa* Types	SCC*mec* Types	PVL Gene	(Ref)
Kuala Lumpur	2003–2008	Hospital	PCR-RFLP *agr* genotyping	NA	NA	NA	No	[25]
Kuala Lumpur Kota Bharu, Selangor Sabah	2006–2008	Hospital	MLST SCC*mec* type.	ST80, ST1284, ST1285, ST1287, ST1288, ST30, ST45, ST22, ST1286, ST101, ST188.	NA	III and IV	Yes	[26]
Kuala Lumpur	2007	Hospital Community	PFGE MLST *spa* typing SCC*mec* typing	ST1, ST239, ST 772.	t037, t074, t127, t421, t657, t3103	III, IIIA and V	Yes	[27]
All Malaysian states	2008	Hospital	PFGE MLST *spa* typing SCC*mec* typing *dru* typing	ST1, ST7, ST22, ST129, ST188, ST1283.	t032, t037, t091, t127, t138, t189, t421, t3213, t4145 t4184, t4213	III, IIIA, IV, V	Yes	[28]
Kuala Lumpur	2008	Hospital	MLST SCC*mec* typing,	ST6, ST22, ST30, ST1178, ST1179.	NA	IV	Yes	[5]
Kuala Lumpur	2003–2008	Hospital	PFGE, MLST, SCC*mec*	ST6, ST22, ST239, ST772, ST1178	NA	III, IV, V	Yes	[29]
Kuala Lumpur	2007–2008	Community	PFGE MLST *spa* typing	ST5, ST1241	t2636	NA	No	[30]
Felda	2009	Community	MLST SCC*mec* typing	ST1, ST188, ST772	NA	V	Yes	[31]
Kuala Lumpur	2009	Hospital	PFGE SCC*mec* virulence genes typing	NA	NA	II, III, IV, V	Yes	[13]
Kuala Lumpur	2010	Hospital	MLST *spa* typing SCC*mec* typing virulence genes typing	ST1, ST7, ST22, ST188, ST239	t032, t037, t127, t138, t189, t421, t932, t2575, t3213, t4150, t4184, t4213	III, IV, V	Yes	[15]
Kuala Lumpur	2012	Hospital Community	PFGE MLST *Spa* typing	ST1, ST3, ST5, ST8, ST121, ST239	t008, t024	NA	Yes	[32]
Kuala Lumpur	2011–2012	Hospital	PFGE MLST SCC*mec* Typing	ST1, ST5, ST6, ST22, ST239, ST508, ST772, ST1137	NA	II, III, IV, IVa, IVb, V	Yes	[33]
Selangor	2012	Hospital	MLST *Spa* typing *dru* typing	ST5, ST6, ST20, ST22, ST80, ST239, ST241, ST573	t002, t032, t037, t304, t363, t421, t458, t860, t1378, t1544, t2029, t4150, t4152, t4184, t6405	IV	No	[34]
Sabah	2012	Community	PFGE MLST *Spa* typing SCC*mec* typing,	ST30	t019	IV	Yes	[35]
Kuala Lumpur	2012	Hospital Community	MLST *spa* typing *agr* genotyping virulence genes typing	ST1, ST3, ST5, ST8, ST121, ST239.	t008 t024	NA	Yes	[32,36]
Selangor	2012–2013	Community	MLST *spa* typing SCC*mec* typing *agr* group typing Virulence genes typing	ST5, ST97, ST548	t026, t145, t159, t186, t336, t548, t701, t1381, t14331, t14503, t6290	I, III	Yes	[37]
Terengganu	2014	Hospital	MLST *spa* typing	ST772	t657		Yes	[38]
Selangor	2015	Community	MLST *spa* typing SCC*mec* typing	ST88, ST96, ST508	t002, t008, t024, t050, t127, t164, t267, t346, t505, t701, t1236, t2883, t4720	I, V	Yes	[39]
Kuala Lumpur	2014–2015	Hospital	MLST SCC*mec* typing agr typing	ST1, ST5, ST6, ST8, ST22, ST45, ST88, ST188, ST239, ST769, ST772, ST1178, ST3547	NA	III IV, Iva, IVc, V	Yes	[40]
Kuala Lumpur	2016	Hospital	MLST SCC*mec* typing *spa* typing	ST239	t037, t041, t032, t138, t421	III	No	[12]
Terengganu	2015–2016	Community	*spa* typing SCC*mec* typing agr	NA	t032, t037, t304, t421, t8696	I, II, III, IV	Yes	[41]
Perak	2016	Hospital	SCC*mec* typing	NA	NA	II, III, IV, V	No	[42]
Selangor	2016	Hospital	*spa* typing	NA	t127, t223, t790, t2246	NA	No	[43]
Kuala Lumpur	2018	Hospital	PFGE MLST, SCC*mec* typing	ST1, ST5, ST6, ST22, ST30, ST45, ST152, ST239, ST772, ST951, ST1179	NA	II, III, IV, V	Yes	[9]

NA: Not applicable.

**Table 2:** Frequencies of multilocus sequence typing (MLST) and staphylococcal protein A (spa) and SCCmec types among MRSA in Malaysia from previous publications (2002–2020).

*Spa*		MLST (ST)		SCC*mec*	
Types	N (%)	Types	N (%)	Types	N (%)
t002	2 (14.3)	ST1	9 (47.4)	I	3 (16.6)
t008	3 (21.4)	ST3	2 (10.5)	II	5 (27.8)
t019	1 (7.1)	ST5	7 (36.8)	III	13 (72.2)
t024	3 (21.4)	ST6	6 (31.6)	IIIA	2 (11.1)
t026	3 (21.4)	ST7	2 (10.5)	IV	13 (72.2)
t032	5 (35.7)	ST8	3 (15.8)	IVA	2 (11.1)
t037	6 (42.9)	ST20	1 (5.3)	IVB	1 (5.5)
t041	1 (7.1)	ST22	8 (42.1)	IVC	1 (5.5)
t050	1 (7.1)	ST30	4 (21)	V	11 (61.1)
t074	1 (7.1)	ST45	3 (15.8)	-	-
t091	1 (7.1)	ST80	2 (10.5)	-	-
t0127	5 (35.7)	ST88	2 (10.5)	-	-
t138	3 (21.4)	ST96	1 (5.3)	-	-
t145	1 (7.1)	ST101	1 (5.3)	-	-
t159	1 (7.1)	ST121	2 (10.5)	-	-
t164	1 (7.1)	ST129	1 (5.3)	-	-
t186	1 (7.1)	ST152	1 (5.3)	-	-
t189	2 (14.3)	ST188	5 (26.3)	-	-
t223	1 (7.1)	ST239	10 (52.6)	-	-
t267	1 (7.1)	ST241	1 (5.3)	-	-
t304	1 (7.1)	ST508	2 (10.5)	-	-
t346	1 (7.1)	ST573	1 (5.3)	-	-
t363	2 (14.3)	ST769	1 (5.3)	-	-
t421	6 (42.9)	ST772	7 (36.8)	-	-
t458	1 (7.1)	ST951	1 (5.3)	-	-
t505	1 (7.1)	ST1137	1 (5.3)	-	-
t548	1 (7.1)	ST1178	3 (15.8)	-	-
t657	2 (14.3)	ST1179	2 (10.5)	-	-
t701	2 (14.3)	ST1241	1 (5.3)	-	-
t790	1 (7.1)	ST1283	1 (5.3)	-	-
t860	1 (7.1)	ST1284	1 (5.3)	-	-
t932	1 (7.1)	ST1285	1 (5.3)	-	-
t1236	1 (7.1)	ST1286	1 (5.3)	-	-
t1378	1 (7.1)	ST1287	1 (5.3)	-	-
t1381	1 (7.1)	ST1288	1 (5.3)	-	-
t1544	1 (7.1)	ST3547	2 (10.5)	-	-
t2029	1 (7.1)	-	-	-	-
t2246	1 (7.1)	-	-	-	-
t2575	1 (7.1)	-	-	-	-
t2636	1 (7.1)	-	-	-	-
t2883	1 (7.1)	-	-	-	-
t3103	1 (7.1)	-	-	-	-
t3213	2 (14.3)	-	-	-	-
t4145	1 (7.1)	-	-	-	-
t4150	2 (14.3)	-	-	-	-
t4152	1 (7.1)	-	-	-	-
t4213	1 (7.1)	-	-	-	-
t4184	3 (21.4)	-	-	-	-
t4213	1 (7.1)	-	-	-	-
t4720	1 (7.1)	-	-	-	-
t6290	1 (7.1)	-	-	-	-
t6405	1 (7.1)	-	-	-	-
t8696	1 (7.1)	-	-	-	-
t14331	1 (7.1)	-	-	-	-
t14503	1 (7.1)	-	-	-	-

## Data Availability

Not applicable.
